# Development and validation of a distress measurement for insulin injections among patients with diabetes

**DOI:** 10.1038/s41598-023-38982-1

**Published:** 2023-07-20

**Authors:** Eujin Choi, Min-Sun Kim, Juhee Cho, Sooyeon Kim, Eun Kyung Kwon, Youngha Kim, Danbee Kang, Sung Yoon Cho

**Affiliations:** 1 Department of Pediatrics, Samsung Medical Center, Sungkyunkwan University School of Medicine, 81 Irwon-ro, Gangnam-gu 06351, Seoul, Republic of Korea; 2 Department of Clinical Research Design and Evaluation, SAIHST, Sungkyunkwan University, 81 Irwon-ro, Gangnam-gu 06351, Seoul, Republic of Korea; 3 Center for Clinical Epidemiology, Samsung Medical Center, Sungkyunkwan University School of Medicine, Seoul, Republic of Korea

**Keywords:** Diseases, Endocrinology, Paediatrics, Quality of life

## Abstract

Insulin injections are stressful but necessary for people with diabetes. This study aimed to develop and validate the Distress of Self-Injection (DSI) scale for patients with diabetes aged ≥ 10 years. We created a questionnaire to evaluate DSI after examining each item following a literature review. The DSI scale with 20 questions in three domains (physical [4], psychosocial [7], and process [9]) was developed and tested at the Samsung Medical Center in Seoul, Korea, from April to September 2021. To verify structural validity, exploratory and confirmatory factor analyses (CFA) were conducted. Internal consistency was also calculated. To assess construct and criterion validity, the correlation between the DSI scale and Korean version of the Problem Areas in Diabetes (PAID-K) scale was obtained. Cronbach’s alpha varied from 0.69 to 0.87, and the DSI score was 0.90, demonstrating acceptable internal consistency. CFA fit indices (CFI = 0.980; RMSEA = 0.033) were favorable. DSI and pertinent PAID-K domains correlated strongly. For measuring self-injection distress, the DSI score had good accuracy. For patients with diabetes aged ≥ 10 years who self-inject insulin, the DSI was a viable and accurate method for quantifying discomfort associated with insulin injection. Health practitioners should use the DSI to communicate with patients about their suffering.

## Introduction

The total number of patients with diabetes is expected to increase from 11 million in 2000 to nearly 20 million in 2025 and may increase steadily in the future^1^. In patients with diabetes, self-care activities are performed to maintain glycated hemoglobin (HbA1c) in the target range to prevent delayed onset of devastating complications^2,3^. Regular insulin injection is required for lifelong treatment in patients with type 1 or 2 diabetes not well-controlled by oral medication alone^4–6^. Approximately 31% of diabetes patients are treated with insulin^7^ specifically, 15.4% ± 1.4% use insulin only, while 13.6% ± 11.1% use insulin plus oral medications^8^. Daily multiple injections can be painful and distressing^9^, resulting in poor compliance^10^. This complicates the treatment in childhood, and the condition can progress to microvascular and macrovascular dysfunction and other metabolic disorders. Therefore, researchers have sought reliable methods to measure distress caused by daily injections in patients with diabetes.

While the Problem Areas In Diabetes (PAID) scale is a useful tool for measuring the general emotional distress associated with diabetes^11^, it is not specifically designed to evaluate the distress associated with insulin injections. Furthermore, the 17-item Diabetes Distress Scale and the 28-item Type 1 Diabetes Distress Scale are tools that cover comprehensive distress associated with diabetes but these tools also cannot assess distress associated with self-injection^12,13^. However, insulin injection itself can cause distress to patients with diabetes. Patients feel different pain according to the different stages of insulin injection, such as needle insertion, insulin injection, and post-injection bleeding or bruising^14–17^. Furthermore, both process-related factors such as correct site rotation, disposal of used sharps, and the use of safety devices, and emotional factors such as the fear of being judged or discriminated against when injecting insulin in public, workplace discrimination, and limitations in traveling due to insulin injection also have distressed in patients with diabetes^14,18^. The detailed understanding of the specific aspects of insulin self-injection that patients find most challenging can help healthcare professionals develop tailored interventions that target the specific needs of each patient, ultimately leading to better management of diabetes. Despite these unmet needs, existing instruments have not been identified that can effectively measure them. Although the Insulin Technique Questionnaire was developed to assess daily injection skills and problems, this tool is more focused on measuring the knowledge of insulin injection techniques instead of covering social or psychological issues related to self-injections^19^. Furthermore, the previous measures did not perform the validation studies in children and adolescents. Children and adolescents with diabetes and their parents or caregivers experience psychological or physical problems, and a factor responsible for this burden is insulin injection-related stress^20,21^. Additionally, considering that self-injection issues are more pronounced in childhood and adolescence because of low resilience^22,23^, previous tools may be ineffective for assessing distress caused by self-injections in children or adolescents with diabetes. Therefore, we developed and validated the Distress of Self-Injection (DSI) scale to assess self-injection distress in people aged 10 and above, including children, adolescents, and adults with diabetes.

## Results

### Study participants

All 136 eligible participants, including those under 10 years old, completed the study questionnaire and demonstrated full comprehension of all the items. The mean age of participants was 20.9 ± 6.7 years. The participants included 47.1% of men. Most (89.7%) participants had type 1 diabetes, and 80.9% of participants used insulin pens. The duration of insulin use for type 1 diabetes and diabetes of the other type were 11.4 ± 6.7 years, and 4.92 ± 5.4 years, respectively. The mean duration of diabetes was 10.9 ± 6.9 years. The mean HbA1c level for the last 3 months at the time of performing this study was 7.08% ± 1.08% (54 ± 12 mmol/mol), and diabetes-related complications such as neuropathy, retinopathy, diabetic nephropathy, DM foot were found in 42% of patients (Table 1). The mean age at diagnosis was 9.63 ± 4.7 years for type 1 diabetes and 13.5 ± 2.1 years for type 2 diabetes.

**Table 1:** Characteristics of study participants (N = 136).

Characteristics	Overall N (%)
Age, years (mean ± SD)	20.9 ± 6.7
Gender	
Female	72 (52.9)
Male	64 (47.1)
Employment status	
Yes	50 (36.8)
No	86 (63.2)
Education level	
High school graduate or less	90 (66.2)
University graduate	40 (29.4)
Master or PhD graduate	6 (4.4)
Marital status	
Single	131 (96.3)
Married	5 (3.7)
Type of diabetes	
Type 1 diabetes	122 (89.7)
Others (type 2, type 1.5, type C, etc.)	14 (10.2)
Duration of diabetes, years (mean ± SD)	10.9 ± 7.0
Type of insulin injection	
Disposable syringe	8 (5.9)
Insulin pen	110 (80.9)
Both disposable syringe and insulin pen	10 (7.4)
Insulin pump	8 (5.9)
Duration of insulin injections, years (mean ± SD)	10.9 ± 6.9
Number of insulin injections, per day (mean ± SD)	4.5 ± 1.9
Hemoglobin A1c, (%, mmol/mol)	7.1 ± 1.1(54 ± 12)
Complication (N = 58)	
Neuropathy	37 (27)
Retinopathy	18 (13)
Diabetic nephropathy	2 (1.4)
DM foot	1 (0.7)

Values were presented n (%) or mean ± SD.

*SD* standard deviation, *DM* Diabetes mellitus.

**Figure 1:**
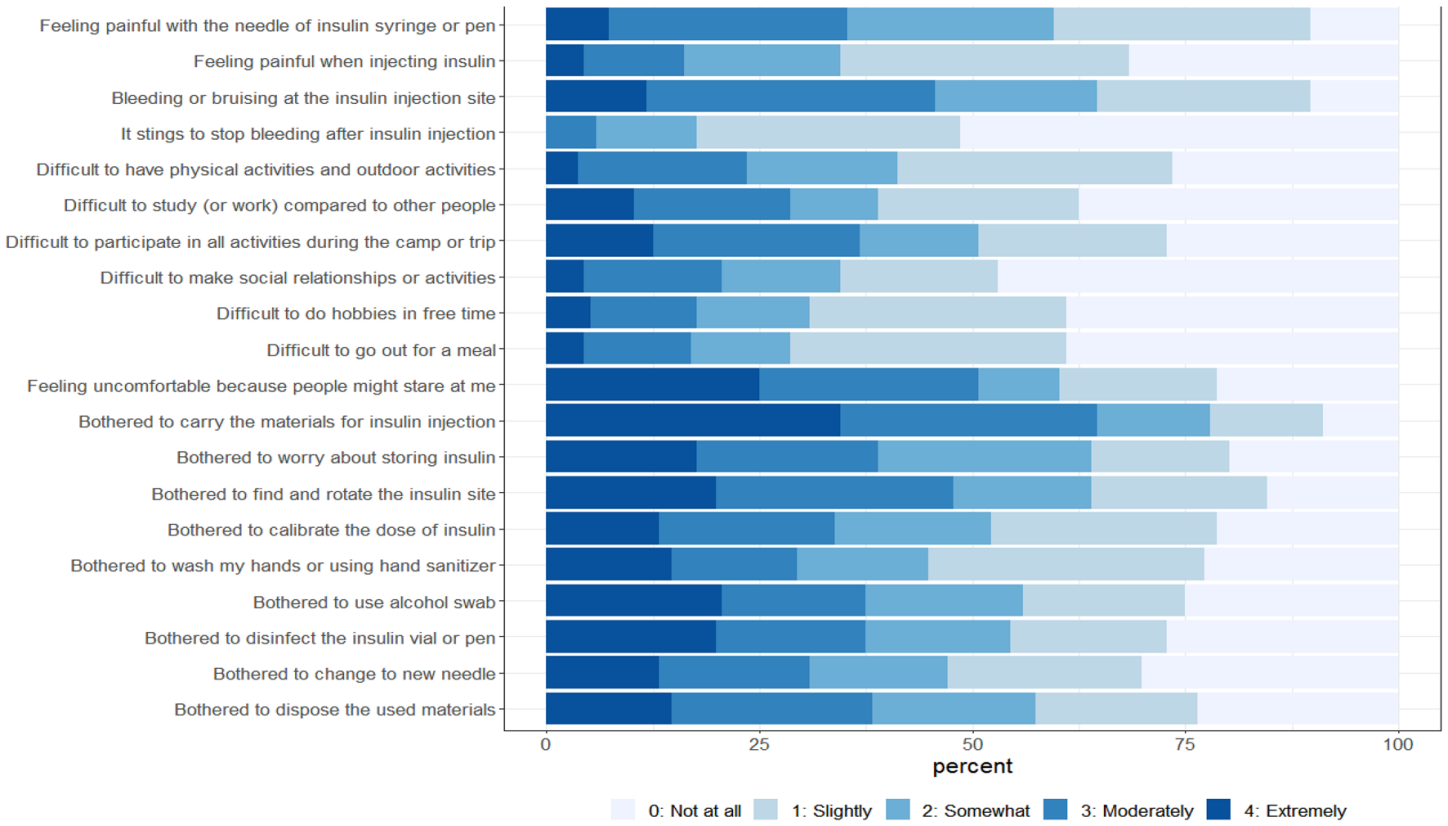
Distributions of distress of self-injection (DSI).

The highest score for distress items was *“Bothered to carry the materials for insulin injection”* followed by *“Bothered to find and rotate the insulin site”* and *“Feeling uncomfortable because people might stare at me”* (Fig. 1).

### Structural validity: exploratory and confirmatory factor analysis

All 23 items satisfied Bartlett’s test for sphericity (*p* < 0.01) and the KMO test for sampling adequacy (*p* = 0.88). EFA indicated a three-factor solution with an eigenvalue > 1.0; thus, it was initially designed for a three-factor solution. In addition, three items (*“concerned with cost for paying insulin,” “The skin at the injection site is hard,” and “There is skin irritation due to the use of alcohol swabs when injecting insulin.”*) were excluded because of lack of significant impact on the interpretable factor solution. Subsequently, *“Bleeding or bruising at the insulin injection site”* (r = 0.39) and “*It stings to stop bleeding after insulin injection”* (r = 0.37) had relatively low factor loading values; however, we did not exclude these items based on the expert opinion that they help quantify physical distress due to insulin injection (Table 2). In addition, *“Feeling uncomfortable because people might stare at me,” “Feeling bothered to worry about storing insulin,” “Feeling bothered to find the insulin injection site and rotate the site,”* and *“Feeling bothered to calibrate the dose of insulin before injections”* showed moderate correlations in both psychosocial and process domains. Thus, we assigned the item to a domain that showed a relatively high correlation.

**Table 2:** Factor loadings from the exploratory factor analysis and reliability of the distress of self-injection dimensions.

No. items (theme)	Factor			Cronbach’s a
	1	2	3	
Physical				0.69
Feeling pain when injecting needle of insulin syringe or pen			**0.76**	
Feeling pain when injecting insulin			**0.77**	
Bleeding or bruising at the insulin injection site			**0.39**	
A stinging sensation is experienced while stopping bleeding after insulin injection			**0.37**	
Psychosocial				0.86
It is difficult to				
Perform physical activities and outdoor activities due to insulin injections	**0.66**			
Study (or work) as much as you want compared to other people due to insulin injections	**0.70**			
Participate in all activities during camping or a trip due to insulin injections	**0.61**			
Form social relationships or engage in activities with the peers due to insulin injections	**0.75**			
Engage in hobbies due to insulin injections	**0.80**			
Go out for a meal due to insulin injections	**0.62**			
Feeling uncomfortable because people might stare at me	**0.50**	0.32		
Process				0.87
Feeling concerned about…				
Carrying the materials for insulin injection when going out		**0.53**		
Storing insulin	0.43	**0.50**		
Finding the insulin injection site and rotating the site	0.41	**0.48**		
Calibrating the dose of insulin before injections	0.39	**0.77**		
Washing my hands or using hand sanitizer before insulin injections		**0.76**		
Using alcohol swab before insulin injections		**0.85**		
Disinfecting the rubber stopper of an insulin vial or pen before insulin injections		**0.83**		
Changing to a new needle of an insulin syringe or pen		**0.56**		
Disposing the used materials (needles, alcohol swabs, etc.) after insulin injections		**0.49**		

The largest loading values were in bold.

**Figure 2:**
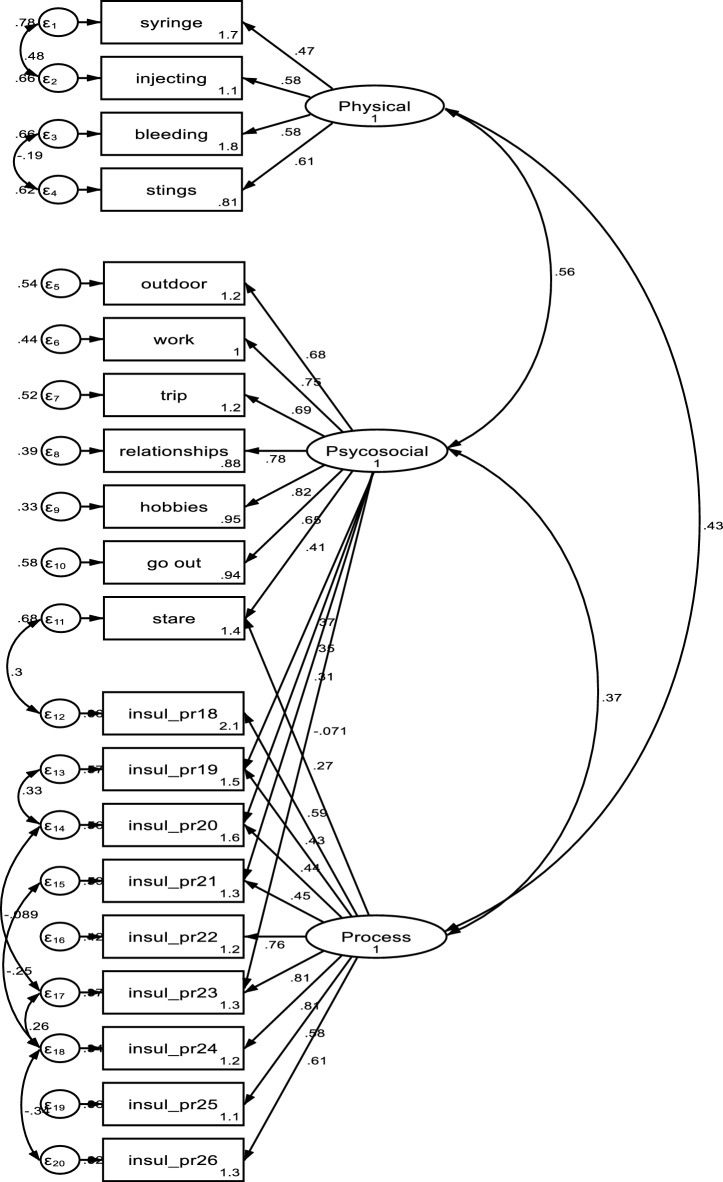
Confirmatory factor analysis of distress of self-injection (DSI).

CFA was used to examine the factor structure of the 20-item DSI scale, which revealed high loadings (0.43–0.79). Although some items, such as *"Bleeding or bruising at the insulin injection site"* and *"Stinging sensation from using an alcohol swab to stop bleeding after insulin injection,"* had relatively low factor loadings, we chose to retain them in the questionnaire due to their content validity, which has been confirmed by experts. To address the issue of low convergence, we searched for items with a modification index value of 10 or higher and incorporated modification indices into the CFA model. The modified model resulted in a reasonable fit for the overall model, with a CFI of 0.980 and an RMSEA (Root mean square error of approximation) of 0.033 (as shown in Fig. 2) obtained after we reconstructed the model using these modification indices.

### Internal consistency

Cronbach’s alpha was 0.90 for the total DSI and ranged from 0.69 to 0.87 for the three subscales, indicating satisfactory internal consistency (Table 2).

### Construct validity and criterion validity

We have calculated the scores for each domain by summing up the scores for each item, and then aggregated these domain scores to calculate a total score out of 80 points. The total DSI score correlated moderately with the PAID-K total score (*r* = 0.58), life interference (*r* = 0.60), and injection stress (*r* = 0.64). In addition, psychosocial and process in the DSI score correlated moderately with the PAID-K total score, life interferences due to injection, and stress due to injection. However, the physical domain of the DSI score correlated weakly with the PAID-K score (Table 3).

**Table 3:** Correlation between distress of self-injection and Korean version of problem areas in diabetes (PAID-K).

Items (theme)	PAID-K total score	Life interference due to injections	Stress due to injections
Physical	0.23*	0.37*	0.39*
Psychosocial	0.62*	0.60*	0.62*
Process	0.44*	0.45*	0.48*
Insulin stress tool total score	0.58*	0.60*	0.64*

*p < 0.01.

## Discussion

This study showed the DSI is a valid tool and reliable to evaluate diabetes distress due to self-injection in individuals with diabetes over the age of 10 years. The emerged three factors were reflected in three subdomains: physical, psychosocial, and process. The construct validity of the tool was validated by both EFA and CFA, and the response scale was valid. Furthermore, concurrent and discriminant validities were demonstrated by varying degrees of correlation with PAID-K, life interference, and stress due to injection. Taken together, our results provide strong evidence supporting the construct validity and reliability of the DSI.

All participants answered all questions correctly. Considering that over half of the study participants were aged under 18 years, the DSI scale could be a viable method to quantify distress of self-injection regardless of the age or illiteracy rate when applied to children and adolescents. EFA and CFA confirmed our hypothesis regarding the underlying constructs of the DSI, which are physical, psychosocial, and process-oriented. The themes of the three domains were consistent with previously identified problems related to self-injection distress in children and adolescents.

The highest distress was the item “*Bothered to carry the materials for insulin injection,” followed by “Bothered to find and rotate the insulin site*” and “*Feeling uncomfortable because people might stare at me.*” These problems have been frequently reported in clinical practice; however, no items that can measure these problems have been reported^19,24^. Thus, the DSI is useful for measuring the psychosocial dimension. Furthermore, our study revealed differences in the physical problem domain by process. Among the patients, 45.6% and 35.3% reported moderate to extreme problems with bleeding or bruising at the insulin injection site and feeling pain when injecting insulin, respectively. On the other hand, only 5.9% of patients reported moderate to extreme problems with bleeding cessation after insulin injection. If patients report pain during needle insertion, techniques such as numbing the injection site or using a smaller needle can be employed. Similarly, if patients experience bleeding or bruising, adjustments to injection technique or site can be made. By addressing these specific areas of distress, the DSI scale can be used to improve patient comfort, adherence to insulin therapy, and ultimately achieve better diabetes management outcomes.

In this study, the internal consistency of the scale was high. CFA also confirmed our hypothesis for DSI constructs, except for two items in the physical function subdomain: *“Bleeding or bruising at the insulin injection site”* and “*It stings to stop bleeding with an alcohol swab after insulin injection.”* These items had relatively weak correlations with the other items and showed low factor loading values in EFA. The weak correlation may be related to the time of distress onset. These two items were related to distress after injections, while the other items were related to distress during the injection^25^.

In terms of construct validity and criterion validity, the total score of DSI and the scores for each subdomain were correlated moderately with PAID-K, life interference, and insulin injection stress. Physical distress in the DSI score showed weak or no correlation with PAID-K, life interference, and stress due to insulin injections. Because PAID-K evaluates only emotional distress due to diabetes, it cannot address physical distress. However, since physical problems from insulin injections also occur frequently, the DSI would be more useful in evaluating these problems^25^.

This study had several limitations. First, we used convenience sampling methods to recruit participants from a single hospital setting, which may have introduced some bias into our sample. Notably, the majority of the patients we enrolled were type 1 diabetes patients who regularly attended outpatient visits and followed up with counseling. However, we emphasize that the development of our study questionnaire involved extensive discussions among experts and careful consideration of previous literature on the topic. Previous studies have shown that patients with diabetes experience similar problems related to self-injections^25^. Although further research is needed, the DSI scale could be a reliable measure for patients with diabetes in other countries. Second, no test–retest analysis was performed. Our study design did not include any clinical stability evaluation. However, the items included in the DSI were relatively objective measures. Third, to reduce the respondents’ distress, the convergent validity of the DSI scale’s physical or process distress subscale was not investigated using separate instruments.

Our tool will help patients specifically pinpoint which parts of the insulin injection steps they are having difficulty with. We also think that this tool will contribute to increasing global knowledge of the steps that are difficult. Furthermore, we think that by addressing the causes of the most difficult point, we can improve adherence with insulin use, which will help control diabetes. Recently, many devices have been introduced for insulin treatment, such as continuous subcutaneous insulin infusion, tubeless attachment pumps, and automatic injector therapy. The DSI would be a useful tool to evaluate the efficacy of these devices in measuring the decreasing distress of self-injections in future clinical trials. In addition, the DSI could be helpful in measuring distress due to other self-injection agents (e.g., growth hormone, hemophilia treatment, and anticancer agents)^26–28^.

In conclusion, our study confirmed that the DSI is a valid and reliable scale for quantifying distress of insulin self-injections, including physical, psychosocial, and process aspects. It can be used to assess the distress of insulin self-injections in patients with diabetes, including children and adolescents aged 10 years and older. Health professionals are recommended to use the DSI scale as a tool to communicate with patients about distress of self-injections.

## Methods

### Instrument development

An expert team of two nurses, two pediatricians, three behavioral scientists, and one librarian conducted a thorough literature review before developing a questionnaire to assess DSI. We identified 11 studies related to the measurement tool for distress caused by insulin use in patients with diabetes. Eighteen of 296 items extracted from the literature were retained, and 14 items were additionally selected based on the opinions of expert reviewers. The 32 items were divided into the following domains: general (5 items), physical (6 items), physical society (10 items), process (10 items), and finance (1 item). Two pediatricians reviewed the items and removed general and financial domains from the tool to increase content validity.

Following the qualitative study, 26 items assessing self-injection distress were chosen from physical, psychosocial, and process domains. We then developed a Korean translation of the English version. Respondents were asked to rate each statement on a 5-point Likert scale (0 = not at all; 1 = a little; 2 = slightly; 3 = quite a bit; and 4 = very much). Sentences were made simple and easy to understand because the DSI scale is intended to cover children as well as adolescents and adults.

To perform content validation, we used modified Delphi methods. Initially, we identified a comprehensive list of items and built consensus from the feedback provided by expert participants in the preceding rounds. The modified Delphi method consisted of two rounds of email questionnaires and a final face-to-face meeting. This method has been described in previous studies^29^. Three of the 26 questions *("How satisfied are you with the type of injection?", "How much do you think insulin injections interfere with your daily life?",* and *"How stressful are insulin injections for you?”*) covered overall distress rather than a single domain and were discarded by content experts in the first round. The other 3 questions (*“Are you concerned about the cost of purchasing drugs and materials used for insulin injection?”, “The skin on the injection site is hard or lumpy.”,* and *“There is skin irritation caused by the use of alcohol during insulin injection.”*) were related to an economic state or an outcome of an insulin injection, therefore those were discarded. Finally, a set of 20 items divided into three domains (physical [4], psychosocial [7], and process [9]) was provided to the study participants.

### Psychometric validation

#### Study participants

We conducted a cross-sectional study of insulin-treated patients with diabetes aged 10–40 years who had visited the department of pediatrics at Samsung Medical Center in Seoul, Korea, from April to September in 2021. We recruited the patients who were confirmed with diabetes and were using insulin self-injection for control of diabetes using convenience sampling methods during the study period. We excluded patients who had any physical or psychiatric condition that would interfere with completing the questionnaire. When eligible patients visited the clinic, we explained this study and obtained informed consent. If the patients were aged under 19 years, the consent of the parent as a legal representative was obtained, and the consent of the child was obtained using a separate child consent. The study protocol was approved by the Institutional Review Board (IRB) at the Samsung Medical Center in Seoul, Republic of Korea, and all participants provided informed consent (IRB number: 2020-12-156-009 SMC). All methods were performed in accordance with the appropriate guidelines and regulations.

#### Measures

We used the 20 items of draft DSI. In addition, we used the Korean version of the PAID scale (PAID-K) to quantify emotional distress of patients with diabetes and test for concurrent and discriminant validities^30,31^. The PAID-K scale is a 20-item representative self-reported instrument for measuring diabetes-related emotional distress that covers a range of emotional problems in patients with diabetes^32^. It was originally developed in the United States of America for use in patients with diabetes. The validity and reliability of PAID has been well-established in the Korean language^33^.

We collected sociodemographic data, including the educational level, marital status, monthly family income, and current employment status. Clinical data were obtained from electronic medical records, including the years since diagnosis, type of diabetes, and type of insulin injection. After conducting the survey, the research nurses asked the participants if there were any items in the DSI that were difficult to respond to.

#### Statistical analysis

Descriptive statistics of DSI items and participant characteristics are expressed as mean ± standard deviation. We performed Bartlett’s test for sphericity and the Kaiser–Meyer–Olkin (KMO) test for sampling adequacy before the exploratory factor analysis (EFA). A significant statistical test in Bartlett’s sphericity test demonstrated that the correlation matrix was not an identity matrix (rejecting the null hypothesis). The KMO test was used to determine the strength of the partial correlation between variables. KMO values closer to 1.0 are ideal, while those < 0.5 are unacceptable.

To evaluate structural validity, EFA was performed to determine the underlying structure of the DSI^34^. A common factor model with alpha factor extraction was used. Alpha extraction develops factors by identifying item groupings with maximum internal consistency, making it a viable option for instrument development^35^. We also performed the confirmatory factor analysis (CFA) using maximum likelihood with missing values to test whether our factor structure fit the data. Several goodness-of-fit indices were used to evaluate the model fit, including the comparative fit index (CFI), and root mean square error of approximation (RMSEA). A CFI score > 0.9 and RMSEA < 0.08 indicates a good fit to the data^36,37^. Factor loadings in CFA were categorized as low (< 0.30), midrange (0.30–0.59), and high (≥ 0.60)^38^.

To test the internal consistency, Cronbach’s alpha was calculated using derivation and validation data to test the DSI. We expected a value > 0.70, the accepted standard for instrument reliability.

Regarding hypothesis analysis to confirm construct and criterion validity, we assumed that the DSI domain and total scores would moderately correlate with the PAID-K total score, life interference, and injection stress (0.30–0.70).

A *p*-value < 0.05 (two-sided) was used to indicate statistical significance, and all statistical analyses were performed using STATA version 16 (StataCorp LP, College Station, TX, USA) and R 4.1.2. (R Foundation for Statistical Computing, Vienna, Austria).

### Ethical approval

The study protocol was approved by the Institutional Review Board (IRB) at the Samsung Medical Center in Seoul, Republic of Korea, and all participants provided informed consent (IRB number: 2020-12-156-009 SMC).

## Data Availability

The datasets used and/or analyzed during the current study are available from the corresponding author on reasonable request. The data that support the findings of this study are available on request from the corresponding author [SY Cho]. The data are not publicly available due to containing information that could compromise research participant consent.

## Abbreviations

CFA: Confirmatory factor analyses
CFI: Comparative fit index
DSI: Distress of self-injection scale
EFA: Exploratory factor analysis
HbA1c: Glycated hemoglobin
KMO: Kaiser–Meyer–Olkin
PAID: Problem areas in diabetes
PAID-K: Korean version of the problem areas in diabetes
R: R foundation for statistical computing, Vienna, Austria
RMSEA: Root mean square error of approximation
SRMR: Standardized root mean squared residual
STATA: StataCorp LP, college station, TX, USA
